# Gene Expression Predicts Histological Severity and Reveals Distinct Molecular Profiles of Nonalcoholic Fatty Liver Disease

**DOI:** 10.1038/s41598-019-48746-5

**Published:** 2019-08-29

**Authors:** Stephen A. Hoang, Abdul Oseini, Ryan E. Feaver, Banumathi K. Cole, Amon Asgharpour, Robert Vincent, Mohammad Siddiqui, Mark J. Lawson, Nathan C. Day, Justin M. Taylor, Brian R. Wamhoff, Faridoddin Mirshahi, Melissa J. Contos, Michael Idowu, Arun J. Sanyal

**Affiliations:** 1 HemoShear Therapeutics, LLC, Charlottesville, Virginia USA; 2 Division of Gastroenterology, Hepatology and Nutrition, Department of Internal Medicine, Virginia Commonwealth University School of Medicine, Richmond, VA USA; 3 Division of Surgical Pathology, Department of Pathology, Virginia Commonwealth University School of Medicine, Richmond, VA USA

**Keywords:** Data integration, Non-alcoholic fatty liver disease

## Abstract

The heterogeneity of biological processes driving the severity of nonalcoholic fatty liver disease (NAFLD) as reflected in the transcriptome and the relationship between the pathways involved are not well established. Well-defined associations between gene expression profiles and disease progression would benefit efforts to develop novel therapies and to understand disease heterogeneity. We analyzed hepatic gene expression in controls and a cohort with the full histological spectrum of NAFLD. Protein-protein interaction and gene set variation analysis revealed distinct sets of coordinately regulated genes and pathways whose expression progressively change over the course of the disease. The progressive nature of these changes enabled us to develop a framework for calculating a disease progression score for individual genes. We show that, in aggregate, these scores correlate strongly with histological measures of disease progression and can thus themselves serve as a proxy for severity. Furthermore, we demonstrate that the expression levels of a small number of genes (~20) can be used to infer disease severity. Finally, we show that patient subgroups can be distinguished by the relative distribution of gene-level scores in specific gene sets. While future work is required to identify the specific disease characteristics that correspond to patient clusters identified on this basis, this work provides a general framework for the use of high-content molecular profiling to identify NAFLD patient subgroups.

## Introduction

Nonalcoholic fatty liver disease (NAFLD) affects a quarter of the adult population and is a leading cause of liver-related morbidity and mortality^1^. This condition is defined mainly by its histology and consists of two principal phenotypes which include a fatty liver and steatohepatitis^2^. Nonalcoholic steatohepatitis (NASH) is characterized by steatosis, inflammation and hepatocellular ballooning which are predominantly seen in zone III of hepatic lobules^3^. NASH has a greater likelihood of progression to cirrhosis than nonalcoholic fatty liver (NAFL)^4^. Current disease models predict a two- to three-fold increase in the population burden of cirrhosis and end stage liver disease due to NASH by 2030^5,6^.

The development of knowledge in NAFLD has been anchored to the histological assessment of the disease. Two principal concepts in this assessment are disease activity and fibrosis stage. Disease activity represents the factors driving the fibrogenic remodeling of the liver towards cirrhosis and is captured by the NAFLD activity score (NAS), which is the sum of the histological severity scores for steatosis, lobular inflammation, and hepatocellular ballooning^3^. On the other hand, fibrosis stage reflects the actual progression towards cirrhosis and is related to clinical outcomes^7^. A substantial body of literature has identified a multitude of metabolic, cell stress, death, inflammatory and fibrogenic pathways that underlie these histological manifestations of disease activity and stage^8^. These have provided numerous targets for therapeutics which have been translated into over 200 active clinical trials for NASH (www.clinicaltrials.gov).

There are unfortunately no drugs yet approved for NASH. Several agents have failed altogether and even the drugs that are now in pivotal trials led to resolution of NASH or regression of fibrosis in less than half the individuals who received these agents in phase 2B trials^9–12^. The reasons for this suboptimal performance are not fully understood. This gap in knowledge is a barrier towards development of more successful therapeutic approaches including the ability to identify which patient may respond best to which therapy, which we begin to address herein.

A potential explanation for the limited clinical success of therapeutics is that the biological processes driving the disease phenotype vary with disease severity, even within the relatively limited range of histological severity included in clinical trials. It is also possible that within diseased populations with similar histological patterns and severity of disease, there may be distinct subpopulations with different disease drivers, as seen with several cancers^13^. Studies of the human transcriptome in NASH have not investigated these possibilities although specific pathways and genes have been linked to disease severity^14–18^. To address this gap, we assessed gene expression profiles along the histological spectrum of NAFLD and used them to develop and validate a gene-level score that reflects histological severity. This scoring methodology enabled us to identify patient subpopulations on the basis of their molecular phenotypes. This demonstration is a necessary first step in establishing a foundation for future development of precision medicine approaches for the treatment of NASH.

## Results

### Identification of gene networks that are regulated across disease activity and stage

We first interrogated the gene expression profile in liver tissue from patients with NAFLD and age- and weight-matched controls. It is important to note that these biopsies were obtained from individuals who were not in a drug treatment trial and were not on any specific NASH drug therapy. The severity of histological features were scored independently by a hepato-pathologist using the NASH CRN scoring system^3^. Supplemental Table 1 summarizes histological activity and stage of the samples in this study with their associated clinical profiles and demonstrate that the cohort had the full histological spectrum of the disease. We applied ordinal regression to identify genes whose expression profiles vary with the severity of the NAFLD activity score (NAS) or fibrosis stage. With a false discovery rate (FDR) threshold of 1%, we observed 2970 differentially expressed genes with respect to disease activity and 1656 genes related to fibrosis stage (Supplementary File 1). The NAS is a composite score that aggregates independent assessments of lobular inflammation, steatosis, and cytological ballooning. Supplementary Fig. 1 shows the distribution of NAS components across the samples. However, very few differentially expressed genes could be associated uniquely with any one of the components, particularly at more stringent FDR thresholds (Supplementary Fig. 2). We also found no evidence of differential expression with respect to assessments of portal inflammation (all FDR-adjusted p-values > 0.9). For these reasons, subsequent analyses focus on the full composite NAS score.

**Figure 1:**
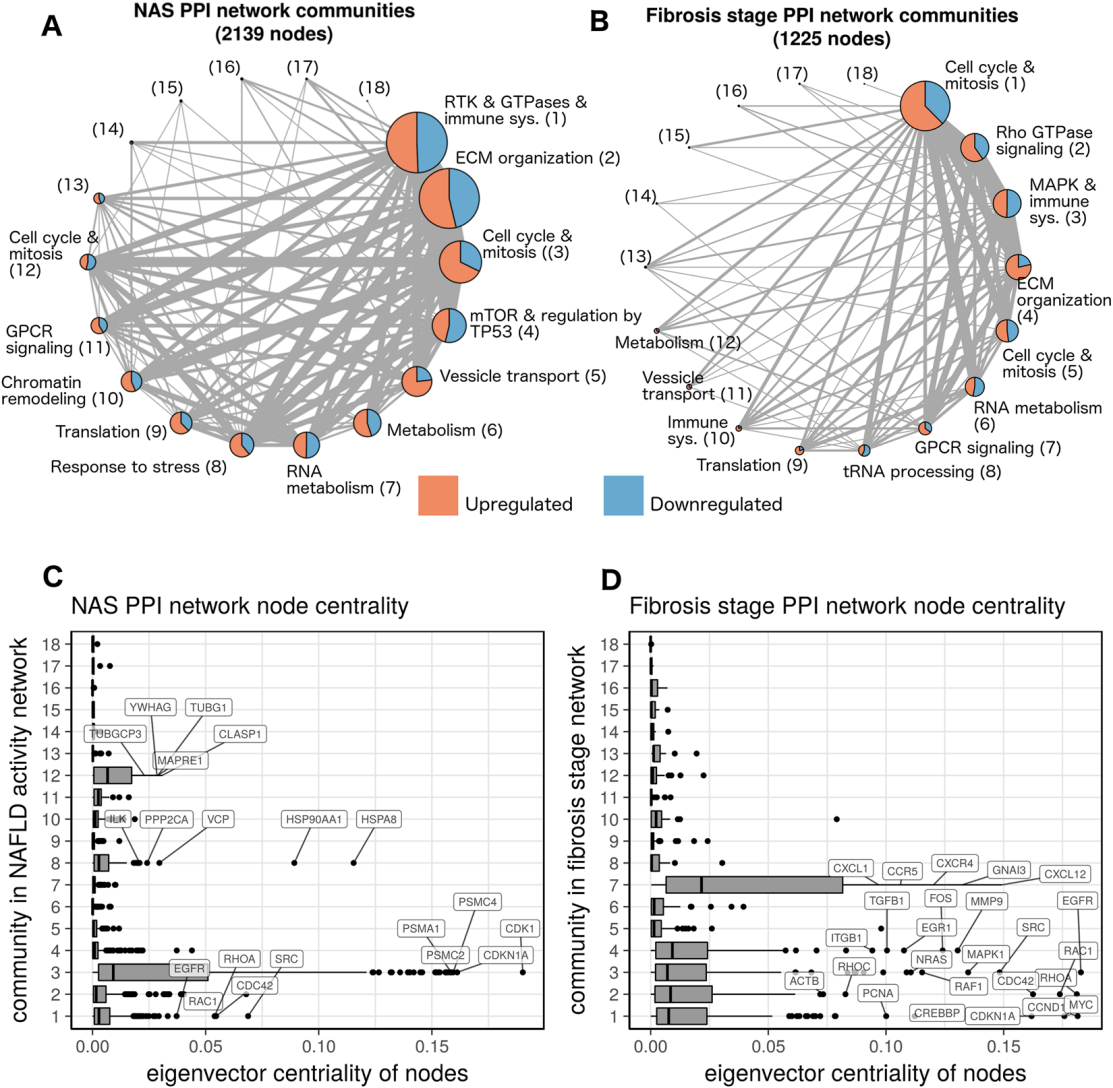
Integration of differentially expressed genes with a protein-protein interaction network highlights hubs involved in the progression of fatty liver disease. (**A**,**B**) A protein-protein interaction network induced by the differentially expressed genes for both NAS and fibrosis stage, respectively. Each node represents a densely connected community of proteins, whose size represents the number of proteins in the community. The node labels provide a summary of the biological processes enriched in each community, as well as a number which is a community identifier. Edge thickness is proportional to the number of connections between communities. (**C**,**D**) Box plots showing the distribution of eigenvalue centrality in the communities of each network. Communities significantly enriched with hubs (nodes with relatively large centrality) are labeled with their top 5 genes by centrality.

The gene expression data were integrated with the STRING protein-protein interaction (PPI) network to identify the portion of the network regulated over the spectrum of the disease^19^. This procedure generated a PPI network where the edges connecting coordinately expressed genes were preserved. The resulting subnetwork, representing the differentially expressed portion of the transcriptome, was further analyzed to identify “communities” and “hubs”. The former are densely connected sets of protein-encoding genes and tend to correspond to biological pathways, while hubs are central to the structure of the network and often represent key regulatory proteins. The resultant networks were differentially regulated with respect to the NAS and fibrosis stage, and are shown in Fig. 1A,B.

Analysis of the NAS PPI network revealed multiple communities, each containing genes that were either up- or down-regulated with respect to increasing NAS (Fig. 1A). The largest community was related to receptor tyrosine kinase (RTK) activity, Rho GTPase signaling, and immune system activation, followed by communities linked to cell cycle and extracellular matrix (ECM) reorganization (pathway enrichment in each community is provided in Supplementary Files 2 and 3). Of note, a relatively small community of genes enriched for metabolic functions was linked to severity of the NAS. The increase in expression of genes involved in cell proliferation (community 3) indicates that tissue repair pathways were also progressively activated with increasing disease activity. As a whole, this network captured many processes that are hallmarks of NAFLD. These processes are coordinately regulated over the spectrum of disease activity, which raises the question of what processes, and more specifically, what genes are the key mediators of this coordination–i.e. what are the hubs in this network and what communities are they in?

In this study, hubs were defined as nodes in the network with high eigenvector centrality. Communities 1, 3, 8, and 12 were significantly enriched for these hub nodes (Bonferroni adjusted p values: 2e-12, 3e-47, 3e-4, and 3e-6 respectively) (Fig. 1C), indicating that specific proteins within these communities likely coordinate patterns of regulation across the entire network. For example, community 3 was strongly enriched for hubs related to proliferation (e.g. CDK1), which are regulators of multiple structural proteins involved in regeneration and tissue repair. The epidermal growth factor receptor (EGFR) also formed a hub within this network suggesting a key role for this gene and related downstream signaling in the repair response to increasing disease activity. A detailed list of genes and their centrality values is provided in Supplementary File 4.

The fibrosis stage network, while smaller than the NAS network, had communities enriched for many of the same processes (Fig. 1B), reflecting the correlation between NAS and fibrosis stage within this predominantly non-cirrhotic population (Supplementary Table 1). Rho GTPase and cell cycle related signaling were the largest communities that were associated with increasing fibrosis stage. Their linkage with both the NAS and fibrosis stage suggest these to be critical pathways linking disease activity to fibrosis progression. Communities 1–4 and 7 were enriched for hubs (Bonferroni-adjusted p-values: 6e-11, 4e-4, 1e-4, 1e-3, and 2e-12), with the G-protein coupled receptor (GPCR) signaling community (community 7) being the most strongly enriched community. Several chemokine genes, such as CXCR4 and CCR5, are among the most central nodes in this community, reflecting an important role for these genes in fibrosis (Fig. 1D). However, the corresponding GPCR-associated community in the NAS network (community 11) was not enriched for hubs, suggesting that GPCR pathways are more relevant for fibrosis severity rather than disease activity.

### Identification of pathways that are differentially expressed with increasing disease activity and fibrosis stage

We next used the Gene Set Variation Analysis (GSVA) which allows the ordinal histological severity score to be regressed against pathway-level abundance values^20^. Whereas the PPI network analysis enabled the discovery of communities of genes that are coordinately regulated, the GSVA analysis allowed us to identify specific, established pathways that are differentially regulated with increasing histological severity. Using both approaches allowed us to identify both the biological pathways perturbed across increasing disease severity as well as the relationship between the processes that are perturbed.

**Figure 2:**
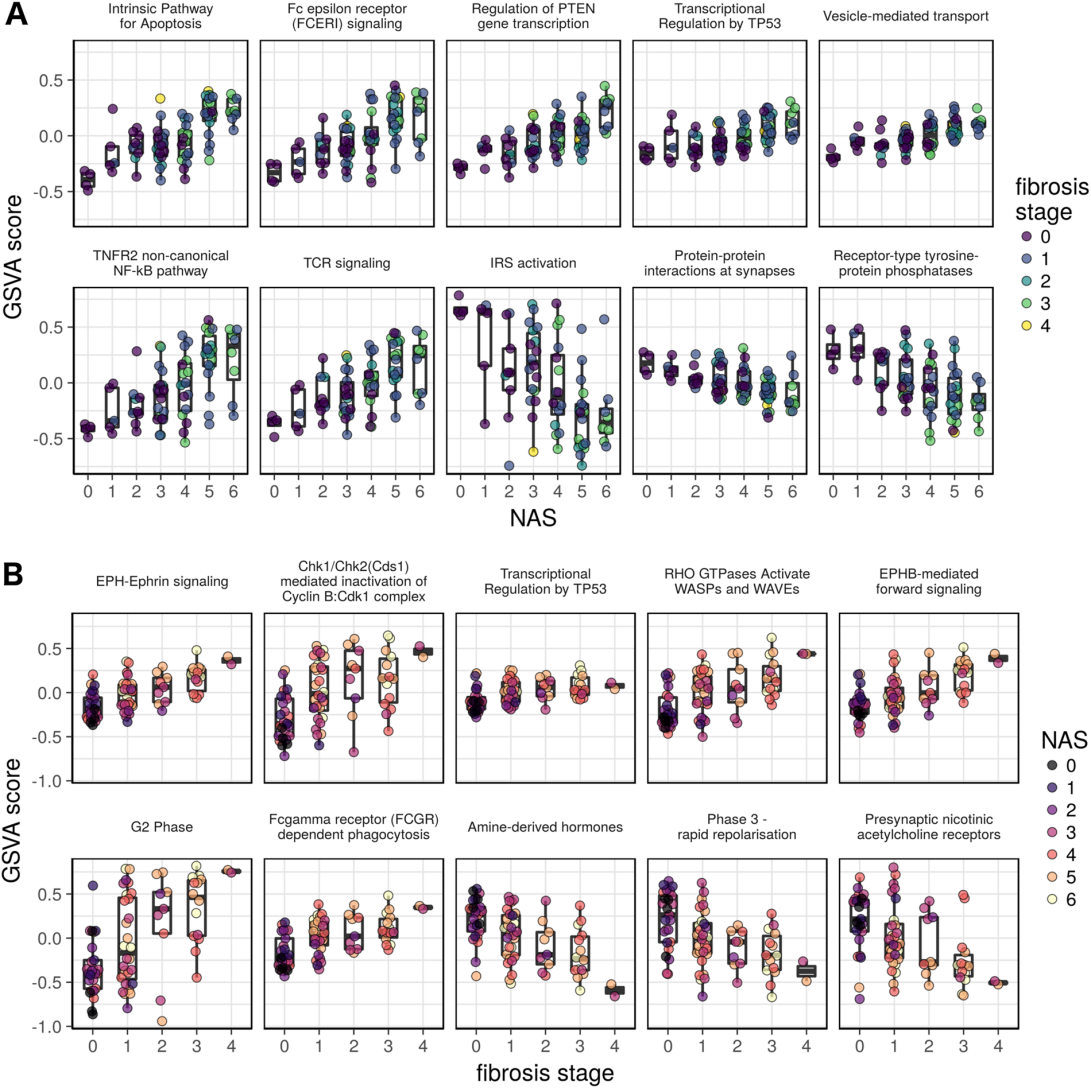
The top Reactome gene sets that are up- and down-regulated with respect to NAS (**A**) or fibrosis stage (**B**). The y-axes represent the GSVA score, which is a pathway-level quantification of gene abundance, and the x-axes represent the clinical assessment. For disease activity (NAS), pathways related to apoptosis, inflammation (Fc epsilon receptor signaling, TNFR2 signaling, T cell receptor (TCR)), cell proliferation (PTEN, TP53) were top pathways whereas for insulin receptor substrate (IRS) signaling pathway was downregulated. For fibrosis, Ephrin signaling related genes were the top pathway while amine derived hormones and nicotinic acetylcholine receptor pathways were down-regulated.

A total of 586 and 392 Reactome pathways were progressively altered (FDR < 1%) with increasing severity of NAS and fibrosis stage, respectively. The full set of pathways identified is provided in Supplementary File 5. The top upregulated pathways, ranked by the strength of their relationship to increasing NAS scores, included mainly those for cell death (intrinsic pathway for apoptosis, programmed cell death), inflammation (Fc epsilon receptor signaling, TNF-receptor 2 noncanonical activation of NFκB, T cell receptor signaling, MHC class II antigen) and cell proliferation (regulation of PTEN, transcriptional regulation of TP53) (Fig. 2A, Supplementary File 5). These indicate a role for both innate and adaptive immune systems as drivers of tissue injury while increasing activation of death pathways reflect the primary mechanisms of hepatocyte loss and cell proliferation pathway activation reflects the liver’s wound healing response to injury. We next performed a similar analysis to identify pathways progressively repressed with increasing disease activity. Not surprisingly, impaired insulin receptor signaling (IRS) was the top pathway downregulated with increasing NAS. Several neuro-signaling-associated pathways, including acetylcholine nicotinic receptor related pathways, were also amongst the top ten down-regulated pathways. Pathway analysis for fibrosis progression revealed ephrin-signaling to be most tightly related to the severity of fibrosis (Fig. 2B). Ephrin receptors are the largest subfamily of receptor protein-tyrosine kinases (RTK) and are known to modulate neural migration, angiogenesis, and oncogenesis^21^. Ephrins signal via the Ephrin receptors (forward signaling) or by alternate pathways (reverse signaling)^22^; specifically, the EphB-mediated forward signaling pathway was activated concordantly with the severity of fibrosis. Rho GTPase signaling pathways, known to modulate oxidative stress, cell migration, phagocytosis, and other cellular processes involving actin reorganization were also closely linked to fibrosis progression^23^. Not surprisingly, the expression of cell cycle, extracellular matrix, and inflammatory signaling pathways were also directly related to the severity of fibrosis (Fig. 2B and Supplementary File 5). Several inflammation and apoptosis related pathways whose activation level was closely related to the NAFLD activity score, e.g. intrinsic apoptosis pathway and Fc epsilon receptor mediated signaling, were also directly associated with fibrosis stage (Supplementary File 5). Interestingly, amine-derived hormone pathway expression was progressively and significantly downregulated with increasing fibrosis stage. Further analysis of this pathway indicated suppression of tryptophan hydroxylase-1 and -2 (FDR-adjusted p-value < 0.01 for both) which are required for serotonin synthesis and dual oxidase-1 (DUOX1) which is a regulator of reactive oxygen species generation^24^. Both serotonin and reactive oxygen species can promote fibrosis and the downregulation of their associated pathways likely reflect adaptations to increased fibrogenic drive.

### Identifying gene expression changes specific to disease activity or fibrosis

The results from the differential expression analysis indicated that while the expression level of some genes was related to the severity of both activity and fibrosis, others were related to either activity or fibrosis exclusively. To further dissect this, we calculated the posterior probability of each gene being uniquely associated with either the NAS or fibrosis stage **(**Supplementary Fig. 3). Genes and pathways most uniquely related to NAS were related to metabolism, respiratory chain electron transport, tricarboxylic acid cycle, and lipid metabolism (Supplementary Fig. 3A,C). This may reflect the upstream metabolic perturbation and fatty acid delivery to mitochondria with increasing disease activity and resultant mitochondrial and electron transport chain activity to generate ATP. On the other hand, it may also reflect the uncoupling of oxidation and phosphorylation and mitochondrial dysfunction that is well known to occur in NASH^25^. Genes uniquely related to fibrosis were enriched for protein translation and ribosomal biogenesis (Supplementary Fig. 3B,D). This likely reflects increased demand for extracellular matrix protein synthesis with increasing fibrosis. Together, these data indicate that while correlated, NAFLD activity and fibrosis stage capture distinct, but overlapping molecular aspects of disease progression.

### Development of gene-level disease scores

Given that the biological processes that determine disease phenotype and progression are dependent on gene expression, we investigated if individual gene expression levels could predict the histological severity of NAFLD. Specifically, we asked two questions: (1) can the histological severity of the disease be inferred from gene expression, and (2) do individual samples show patterns of pathway regulation that signify distinct regulatory profiles? To address these questions we derived gene-level scores that estimate disease severity as a function of gene expression. The scores correspond to severity with respect to NAS or fibrosis stage, and so we refer to them as gNAS (gene-level NAFLD activity score) and gFib (gene-level fibrosis stage) scores.

Ordinal regression models were used to assign a disease progression score for each gene based on its expression for a given patient. The scores were calculated using a 10-fold fitting procedure, where in each fold a set of samples was scored according to models fit to a disjoint set of samples. This procedure simulates a scenario where newly observed samples are scored against a benchmark set of samples. Genes with the highest coefficient of variation of gene-level scores across the dataset convey the greatest information about the relationship between expression and disease severity (see Methods). Thus, we focused on the top 1000 genes based on the coefficient of variation in gNAS and gFib scores. Both gene sets had a 98–99% overlap with differentially expressed genes (FDR 1%).

**Figure 3:**
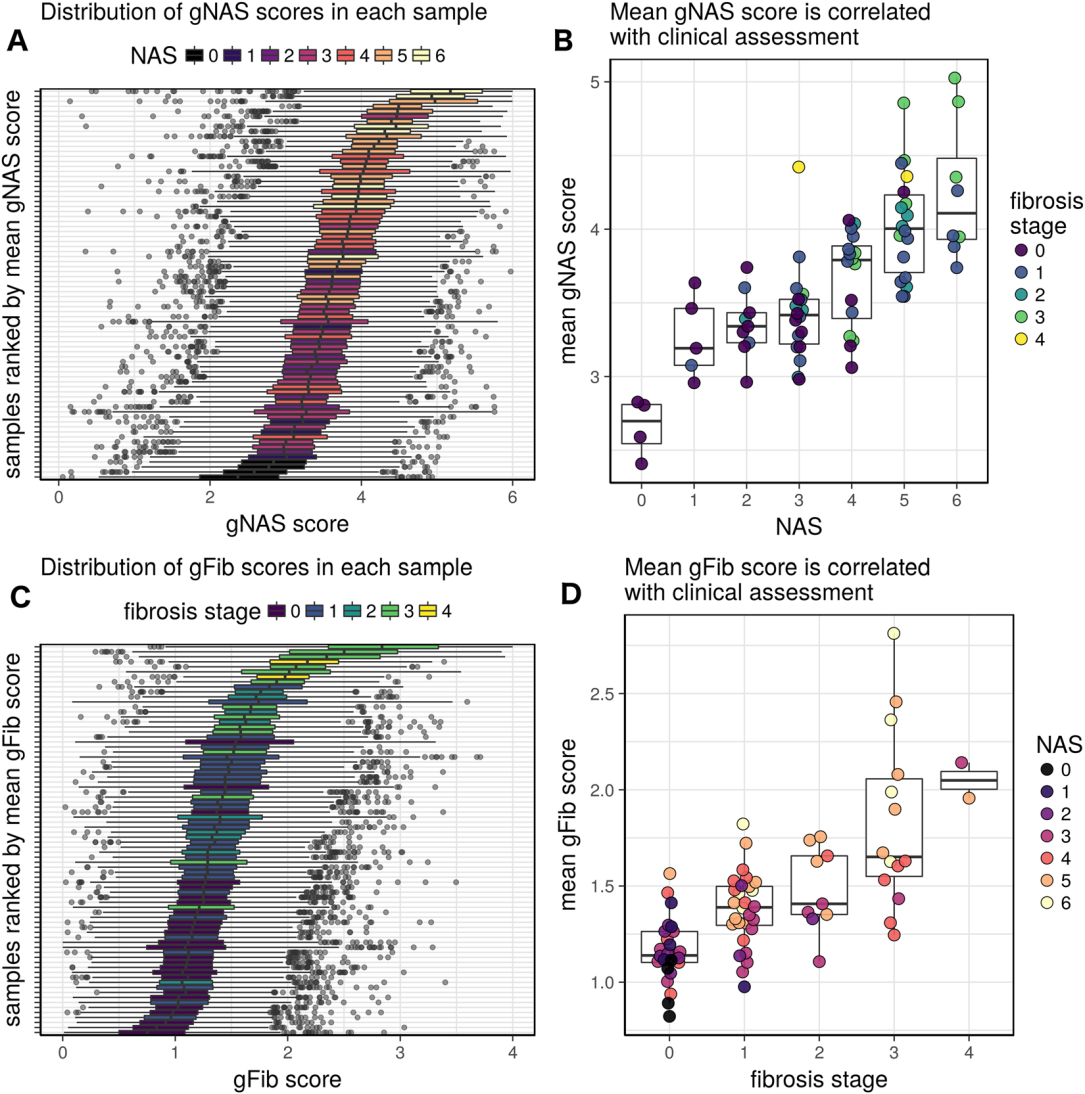
Based on the dynamic range of expression and rank order upon ordinal regression of gene expression levels to the NAFLD activity score (NAS) or fibrosis stage, a gene-level score was derived for all genes tested. The distribution of gNAS scores (**A**,**B**) and gFib scores (**C**,**D**). Plots (**A**,**C**) show the distribution of gNAS or gFib scores for the top 1000 genes in each sample. Plots (**B**,**D**) show the relationship between mean gNAS and gFib scores and histological assessments.

We next ordered the patient samples by mean gNAS and gFib scores from the top 1000 genes and related them to NAS and fibrosis stage respectively (Fig. 3A,C). These gene-level scores demonstrated a strong correlation with histological grade (Fig. 3B,D). This implies that given a benchmark transcriptomic dataset (such as the one presented in this study), the histological severity of a newly observed biopsy sample can be approximated from the expression profile of roughly 1000 genes. In this dataset, the discriminating power of this assessment is greatest at the extremes of the disease spectrum.

**Figure 4:**
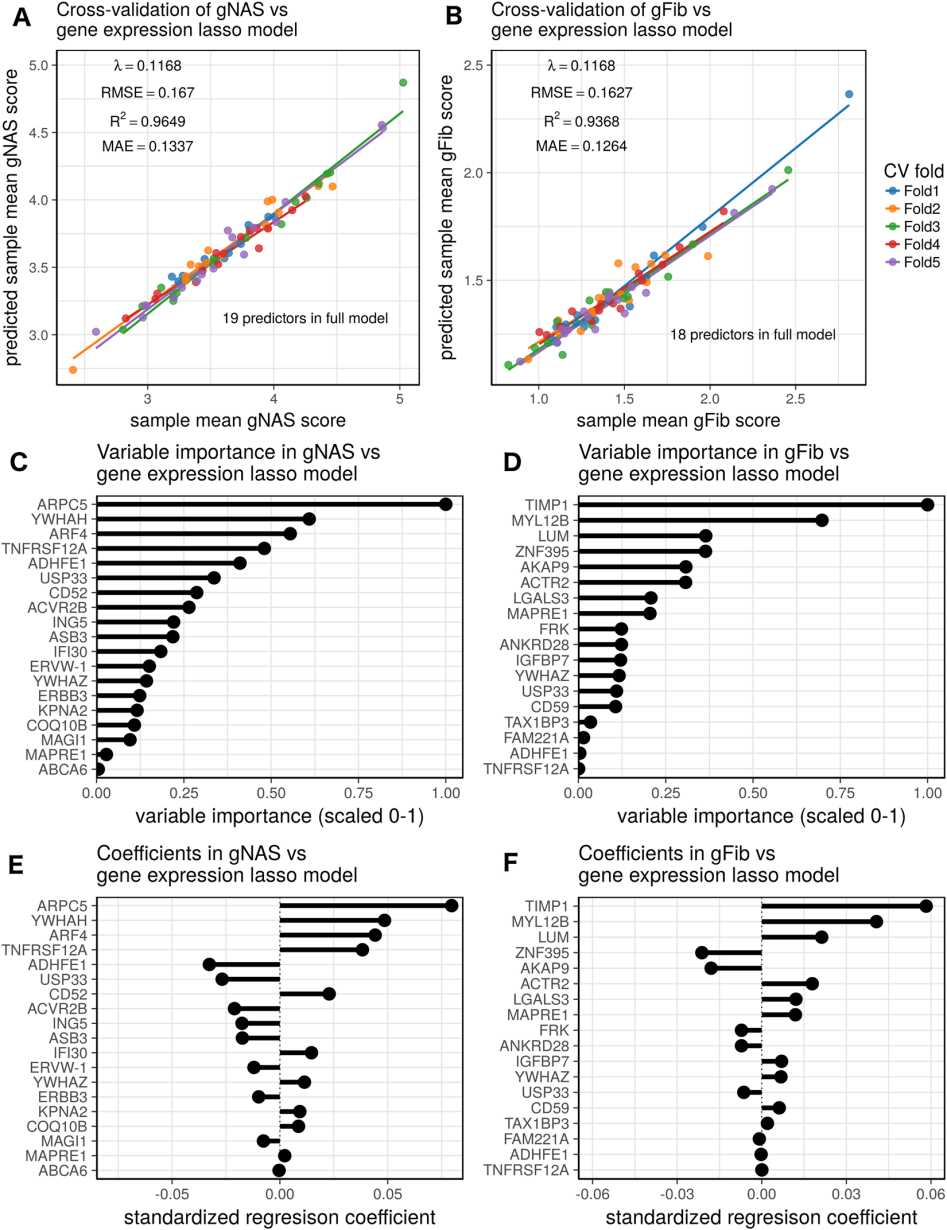
Lasso regression of gene expression values against mean gNAS (**A**,**C**,**E**) or gFib scores (**B**,**D**,**F**). Figures (**A**,**B**) show the results of 5-fold cross-validation for each model, which have 19 and 18 predictors, respectively. The strong performance of the models in cross-validation demonstrates that disease severity can be assessed from the expression levels of a relatively small number of genes. Figures (**C**,**D**) provide the scaled variable importance for model predictors. Figures (**E**,**F**) show the standardized regression coefficients for each model.

To determine if severity can be inferred from the expression of a smaller gene set, we used lasso regression to regress mean gNAS or gFib scores against transcript abundance. Lasso regression was chosen for its ability to perform feature selection and to tune the number of features in the model fit. The regularization parameter was tuned such that roughly 20 predictive genes were selected. While cross-validation RMSE values were better with larger gene sets, the differences were modest (Supplementary Fig. 4). The gNAS and gFib lasso models achieved cross-validated R^2^ values of 0.96 and 0.94, respectively (Fig. 4A,B). These results indicate that the expression levels of a small subset of genes can be used to accurately infer disease severity. The predictor genes span a wide range of biological processes, which includes metabolism, cell-cell interactions, transcription, chromatin dynamics, and transport as well as other processes. The contributions of these specific genes to the model are shown by plotting their variable importance (Fig. 4C,D) and standardized regression coefficients (Fig. 4E,F).

### Identification of distinct gene regulation profiles

The gene-level resolution and continuous nature of these scores enables many possibilities for making fine distinctions in disease progression and for distinguishing between patients with unique transcriptional profiles. If there are distinct molecular subtypes of NAFLD, driven by distinct biological processes, then this would be reflected as distinct patterns in the distribution gene-level scores within samples. For example, a form of NAFLD driven by lipid metabolism would have relatively high scores for genes associated with lipid metabolism. In such a scenario, lipid metabolism would be referred to as a “leading-edge” disease process.

**Figure 5:**
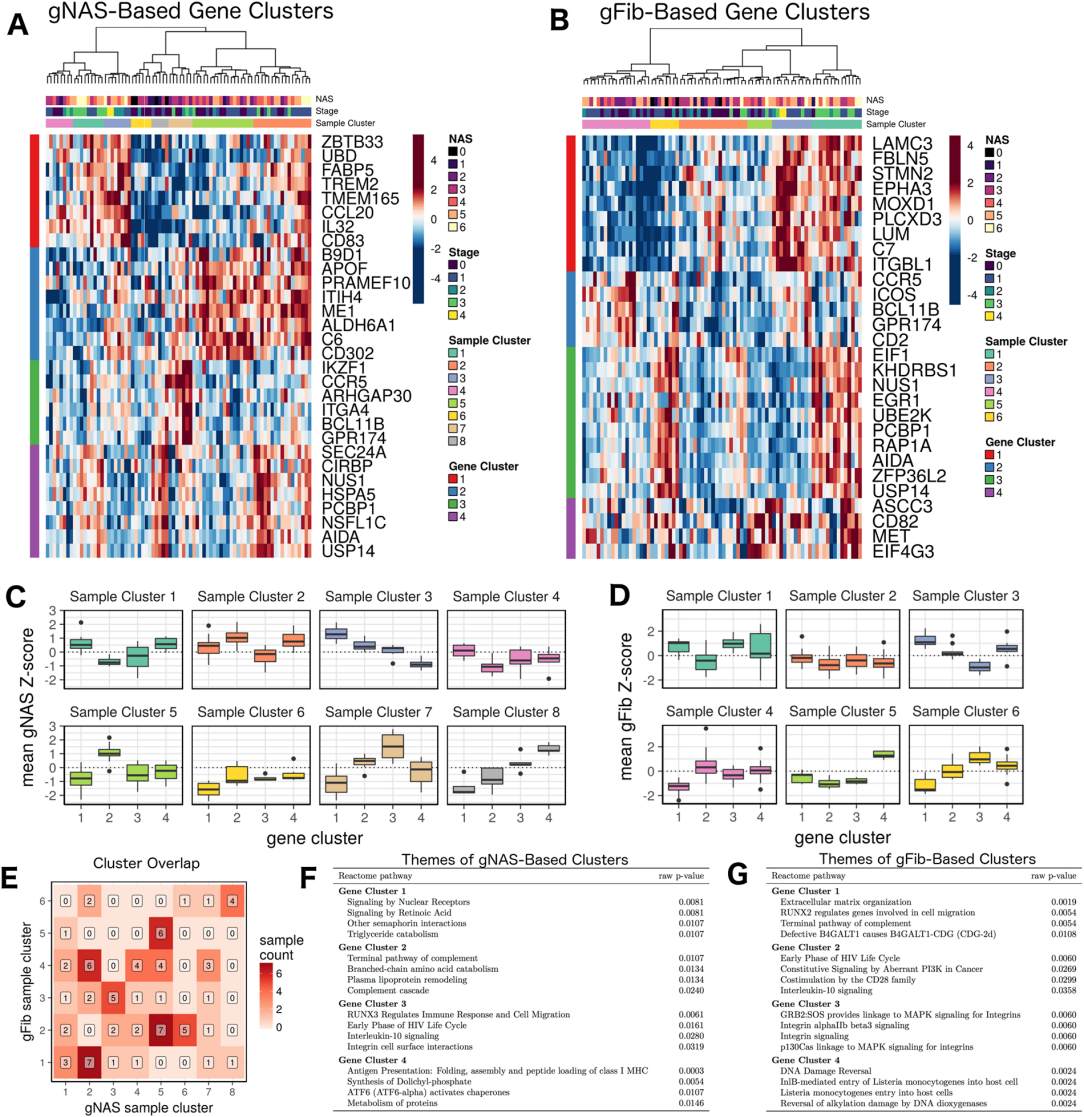
Patterns of gNAS and gFib scores across patient samples reveal distinct molecular profiles. Panels (A,B), respectively, show standardized gNAS and gFib scores across sets of genes that were identified through gene shaving. Sample clusters in these panels show distinct patterns regulation across these genes, and thus represent patients with distinct molecular profiles. Panels (C,D) show the distributions of mean standardized scores for each sample cluster. Within these plots, patterns across gene clusters (x-axis) represent the average molecular profiles of the sample clusters. Panel (E) shows the intersection of the gNAS- and gFib-based sample clusters and provides the number of samples in each cluster pair. Simultaneous consideration of the two partitions provides additional granularity in sample classification. Panels (F,G) show the most strongly represented Reactome pathways in each gene cluster (by Fisher’s exact test). The pathways represented are closely linked to NAFLD progression.

We identified patterns in relative gene-level scores by applying the so-called gene shaving method to the centered and scaled gNAS or gFib scores of the DEGs (1% FDR)^26^. This procedure identified distinct clusters of correlated genes with high variance across samples (Fig. 5A,B). The patterns of gNAS and gFib scores across these genes revealed pronounced sample clusters, which represent groups of patients with distinct, coherent patterns of regulation across the gene clusters (Fig. 5C,D). Examining the overlap between gNAS- and gFib-based clusters provided further granularity in profile distinction (Fig. 5E); i.e. patients in any given gNAS cluster were generally distributed over more than one gFib cluster, and vice versa. The overall functional profiles of both the gNAS- and gFib-based gene clusters highlights pathways that are closely linked to the NAFLD disease process, including ECM remodeling, inflammation, metabolism, integrin signaling, compliment, and DNA damage response (Fig. 5F,G).

**Figure 6:**
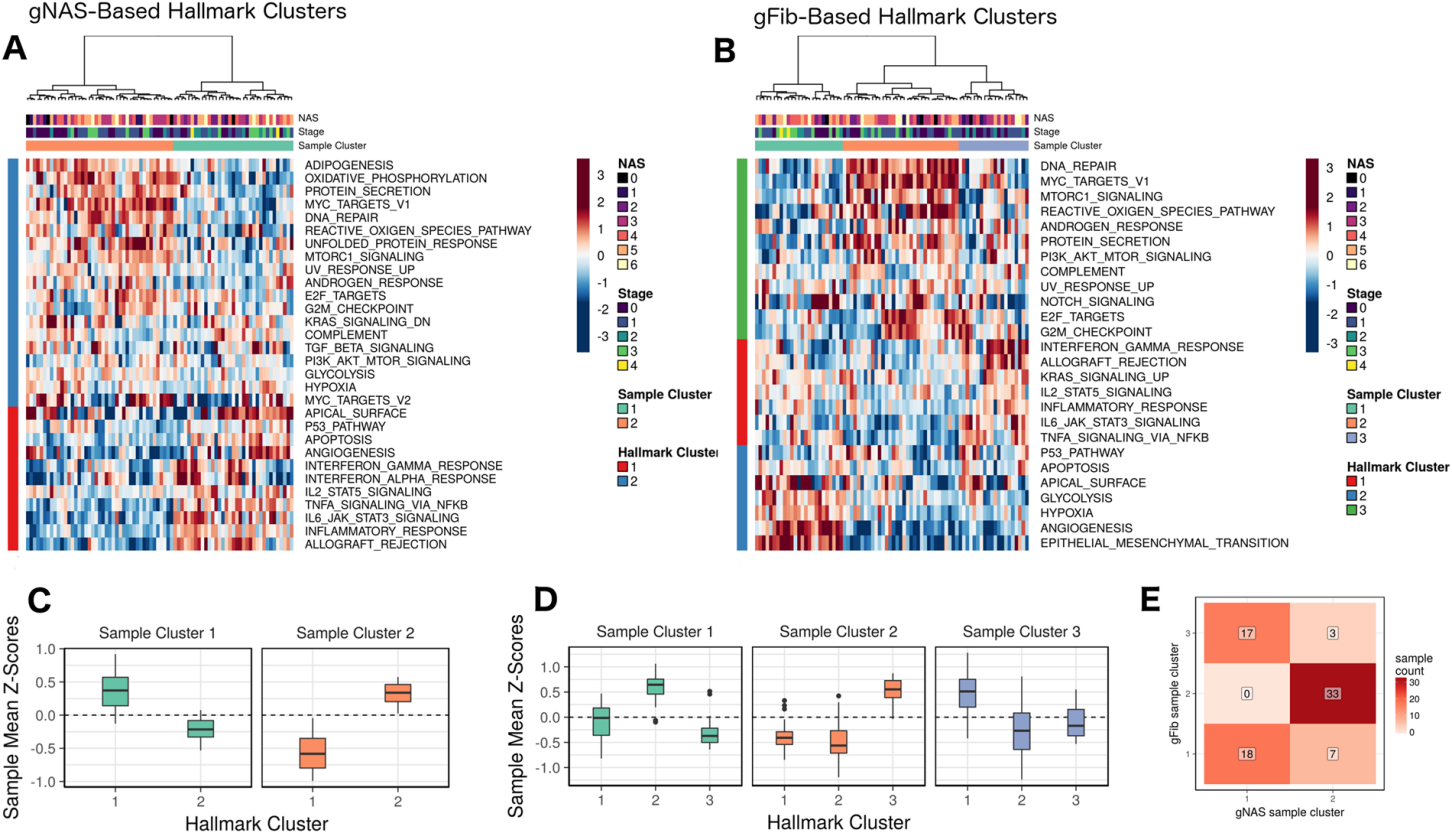
Patterns of pathway-level regulation with respect to gNAS and gFib scores. The heatmaps (**A**,**B**) shows the clustering pattern of samples (columns) and MSigDB hallmark pathways (rows) with respect to mean gene-level scores (values represent column-wise Z-scores). Sample clusters show distinct patterns of pathway-level regulation. Panels (C,D) show the mean sample-wise Z-score in each cluster for the gNAS and gFib analyses, respectively. Higher values in both figures are consistent with relatively advanced disease states. Panel E shows the intersection of the gNAS- and gFib-based sample clusters and provides the number of samples in each cluster pair.

While the previous analysis provides a rational basis for the classification of patient molecular profiles, it is of limited use in identifying leading-edge processes due to relatively small size of the gene clusters. Furthermore, distinct patterns of regulation across the gene clusters may converge on the same pathways. Therefore, we sought to identify pathway-level summaries of variation in gNAS and gFib scores. For this analysis, we selected the so-called hallmark collection of gene sets from the Molecular Signatures Database (MSigDB), since it concisely summarizes a diverse set of biological processes^27^. We computed the mean gNAS and gFib scores of DEGs (1% FDR) in each significantly regulated hallmark gene set (1% FDR). Samples and pathways were then clustered by these values. In the gNAS analysis (Fig. 6A,C), the result shows at least two distinct sample clusters, and two distinct hallmark clusters. Figure 6C summarizes the distinct patterns of pathway-level regulation for each gNAS-based sample cluster. Inflammation and apoptosis (hallmark cluster 1) were leading-edge processes for sample cluster 1. This was not the case for sample cluster 2, which instead implicated cell stress, metabolism, and other pathways as leading-edge processes (hallmark cluster 2). Clusters based on gFib scores show similar patterns (Fig. 6B,D**)**; however, processes associated with morphology and angiogenesis appear as a distinct cluster (hallmark cluster 2).

## Discussion

This study provides a snapshot of the pathways that are transcriptionally regulated in NAFLD and the leading-edge pathways associated with increasing disease activity and fibrosis stage. It also provides insights into how these pathways interact and coordinate activation or suppression with increasingly advanced disease. The development of gene-level scores broadly corresponding to histological severity enables inference of disease phenotypes based on transcriptomic profiles and facilitates a procedure for identifying patients with distinct patterns of gene regulation. The methods presented here are general and can be used to distinguish patients based on any high-content molecular profiling technology. Accordingly, these methods have great potential for furthering research into personalized treatment approaches.

These data not only provide novel insights in to the specific genes and cellular processes driving the disease phenotype in humans (e.g. Ephrin related signaling), but also enable identification of novel drug targets and hypotheses related to disease drivers. A key finding is that inflammatory pathways, including both the innate and adaptive immune systems, are linked to both histological activity as well as fibrosis; a fact which can be potentially leveraged for therapeutics. If further validated, the methods presented in this study for characterizing molecular heterogeneity may serve as a foundation for precision medicine approaches that identify specific disease drivers in a given patient and therapeutically target the pathways relevant to that individual.

The patient clusters identified in this study hint at the presence of distinct molecular subtypes among the patients. This can be inferred from the differences in the leading-edge processes among the patient clusters. A possible biological driver of the clusters is differences in the natural course of the disease. If this is case, the clusters could be interpreted as different patient subtypes with distinct molecular drivers of the disease. A second possibility is that the clusters correspond to snapshots of a dynamic process, in which case the clusters might correspond to various phases of disease progression. Some combination of these two proposals is also possible. However, the lack of association between histological grade and cluster membership suggests that the clusters are driven, at least in part, by something other than disease progression. Future studies will be required to fully elucidate the functional implications of this patient classification strategy. Such studies are important insofar as the establishment of the molecular heterogeneity of NAFLD progression is a priority in the field.

Other studies have investigated gene expression changes that accompany disease progression in NAFLD, notably Wruck *et al*. and Moylan *et al*.^15,28^. The former study is a meta-analysis of several transcriptomic datasets from patient liver biopsies that identifies gene expression changes associated with the progression from NAFLD to NASH. The authors identify several functional pathways and gene sets that are significantly regulated over the course of this transition, which are primarily associated with lipid metabolism. The latter study uses a similar approach, comparing mild (fibrosis stage 0–1) to severe (fibrosis stage 3–4) patients. It identifies a somewhat greater diversity of functional process, which includes several core metabolic subsystems as well as proliferation pathways. Our study also identifies several pathways involved broadly in metabolism and proliferation (Fig. 1 and Supplementary Files 2, 3 and 5); however, our results also demonstrate widespread regulation of inflammatory processes and the extracellular matrix over the course of the disease. There are several possible reasons for the differences observed across these studies, the most obvious of which include differences in patient cohorts and differences in pathway analysis methods. Perhaps a more subtle difference is the fact that this study identifies disease-associated transcriptional regulation by leveraging all of the information contained in the ordinal histological assessments of the disease, whereas the other studies achieve this by binning patients into “early” and “advanced” disease categories. Thus, our approach is likely more sensitive to gene expression changes that correspond to the phenotypic changes that are summarized by histological scoring. Indeed, Pirhaji *et al*. demonstrated that in the case of Huntington’s disease, the use of ordinal regression across a spectrum of disease severity was superior to disease vs. control comparisons in identifying gene expression signatures associated with the disease phenotype^29^.

In addition to studies that further explore the implications of patient classification on the basis of gene expression, this work invites future longitudinal studies that can be used to validate the progressive changes to gene expression observed in these data. Specifically, tracing changes in gene expression in a set of patients over time would be the best validation of the gene-level scoring system and its ability to infer disease progression. Importantly, the development of gene-level activity and fibrosis scores provides a nuanced and content-rich perspective on disease progression, which enables new ways to evaluate central puzzles in the field, such as the placebo response and spontaneous improvement even in the absence of weight loss. Such investigations are expected to provide insights that can be leveraged to develop novel hypotheses and approaches to reverse the disease process.

It is important to note the limitations of this study. We observed relatively few transcriptomic changes uniquely associated with the individual components of the NAS as well as assessments of portal inflammation; however, we observed large effects associated with the composite NAS. There are several potential explanations for this observation. One possibility is that the concomitant progression across all three components of the score may confound analysis of any single component. Additionally, our statistical power for detecting genes associated with the individual NAS components may be limited by the distribution of the scores and/or the sample size. A related limitation of this study is that the patient samples do not uniformly represent the spectrum of the disease. In particular, the majority of the samples represent moderate disease activity (NAS 3–5), with relatively few mild (NAS < 3) and severe (NAS > 5) cases. One likely consequence of this sampling bias is a loss of statistical power; i.e., the number of differentially expressed genes we observed is likely an underestimate. We would also expect a moderating effect on the gene-level scores, since model estimates for the extreme ends of the disease spectrum would be associated with relatively high error. Even so, the relationship between expression levels and gene-level scores is monotonic, so the score rankings should be unaffected. In future studies, these issues can likely be addressed through some combination of alternative statistical approaches and a study design structured with the intent of deconvolving the components of the NAS and/or balancing patient samples over the spectrum of the disease.

There are also a number of potential confounding factors that were either unavailable for this study, or whose inclusion in this analysis was not possible or straightforward given the study design. For example, some patients were taking concomitant medications which may have altered their gene expression profiles. Also, some known NAFLD-associated genetic variants in genes such as PNPLA3 and TM6SF2 could also influence the patient profiles; however, these data were not available.

A broad limitation of this study is that gene expression levels alone may not predict translation into protein or the functionality of the proteins. Ultimately, integrative approaches using larger transcriptomic, proteomic, genomic, and metabolomic data will be needed to build more comprehensive models of disease development and progression. Indeed, some existing studies such as Wruck *et al*. provide high-content data in NAFLD patients that can be used toward this end^30^. These limitations notwithstanding, the current study provides a general framework for leveraging the power of high-throughput molecular profiling to develop precise characterizations of NAFLD development and progression. Similar unbiased frameworks will likely serve as a foundation of future precision approaches for the management of NAFLD.

## Methods

### Study population

Patients presenting with suspected or known NAFLD who were undergoing a standard of care liver biopsy to diagnose and/or to assess the severity of the disease were enrolled in this study. All subjects were enrolled between 2012 and 2016 at a single tertiary care medical center. The study was approved by the institutional review board of Virginia Commonwealth University, and all subjects provided informed consent. All research was performed in accordance with the guidelines and regulations of the review board and the publisher. The liver biopsy was performed using a percutaneous approach or a transjugular approach in all instances. At the time of the biopsy, 1.5–2 cm core of tissue of 16 gauge diameter was sent for histological assessment and 2–5 mm of tissue was snap-frozen in liquid nitrogen at the bedside within five minutes of obtaining the biopsy. Those with biopsy-proven NAFLD were included for this analysis. Control subjects included those who had normal liver histology and did not have evidence of other common etiologies for liver disease such as hepatitis B and C, hemochromatosis, alcohol-associated liver disease. These subjects were either donors for living donor transplant or had a prior history of ALT fluctuations that was evaluated with a liver biopsy.

### Assessment of liver histology

Liver histology was assessed using the NIDDK NASH CRN criteria by two hepato-pathologists^3^. NAFLD was diagnosed by the presence of more than five percent steatosis assessed by histological examination. The nonalcoholic nature of the disease was assessed by clinical history and assessment and by exclusion of an alcohol use disorder using the AUDIT questionnaire^31^. Steatohepatitis was diagnosed by the presence of steatosis along with hepatocellular ballooning and lobular inflammation with or without fibrosis. Those with borderline or definite steatohepatitis were considered together as steatohepatitis for purposes of this analysis. The severity of individual histological features were scored using NASH CRN criteria and disease activity was determined by computing the NAFLD activity score (NAS) which is a composite of the steatosis, inflammation, and ballooning scores. The fibrosis stage was also scored according to the NIDDK NASH CRN staging system from tissue sections stained with Masson’s trichrome stain. Those with stages 1a, 1b, and 1c were considered as stage 1.

### RNA-seq

RNA was extracted from cells using a Qiagen RNeasy RNA Isolation Kit (Qiagen, Gaithersburg, MD) as per manufacturer’s instructions. RNA quantity and quality were assessed using a NanoDrop ND-1000 spectrophotometer (NanoDrop Technologies, Wilmington, DE) and Agilent 2100 bioanalyzer (Agilent Technologies; Santa Clara, CA). cDNA libraries were prepared using a TruSeq Stranded mRNA Sample Preparation kit (Illumina, San Diego, CA). RNA-Seq was performed on the Illumina HiSeq2500 next-generation sequencing platform (Illumina, San Diego, CA).

### Quantification of RNA-seq data

Transcript expression was quantified using the RNA-seq quasi-mapping tool, Salmon, which was run in GC bias-aware mode^32^. Target transcripts were derived from genome assembly GRCh37.75 from Ensembl^33^. Transcript-level quantifications from Salmon were transformed into gene-level count estimates using the tximport R package^34^ and an Ensembl transcript-to-Entrez gene cross-reference derived from Biomart^35^. Genes with low abundance were filtered out of the dataset by applying a minimum expression threshold of greater than 0.5 counts per million (CPM) in at least three samples. Library sizes were adjusted using the TMM normalization method from the edgeR bioconductor package^36,37^. The counts and normalized library sizes were used to transform gene-level counts into log_2_(CPM) values, which were used as the gene-level abundance estimates in subsequent analyses.

### Differential expression analysis

Genes that were differentially expressed across the ordinal spectrum of fibrosis stage or NAS were identified using ordinal regression, which is an approach similar to the one used by Pirhaji *et al*. in Huntington’s disease^29^. Specifically, using the ‘ordinal’ R package, we fit a cumulative link logit model to each gene j:

$$\mathrm{log}\,{\rm{it}}({\rm{P}}(Y\le i|{\hat{x}}_{j}))={\alpha }_{ij}-{\hat{\beta }}_{j}^{T}{\hat{x}}_{j}$$

where i is an ordinal value (i.e. $i\in \{0,1,2,3,4,5,6\}$ for NAS and $i\in \{0,1,2,3,4\}$ for fibrosis stage), Y is the sample score (i.e. the clinical call), and x is a vector of predictors, which in this case is gene expression and the sex of the patient. The two-tailed z-test was used to test the null hypothesis that the gene abundance regression coefficient is equal to zero. The resulting p-values were adjusted across all genes using the Benjamini-Hochberg method. For each gene, we calculated the Bayesian posterior probability of the null hypothesis being false using the method described by Allison *et al*.^38^. This value can be interpreted as the posterior probability that a gene is differentially expressed. Fold changes reported throughout this report correspond to the difference in the mean log_2_ CPM between the top two and bottom two levels of the ordinal range.

### Protein-protein interaction network analysis

The differential expression results were integrated with the human STRING v10 protein-protein interaction network^19^. The network was obtained using the ‘STRINGdb’ R package from Bioconductor^39^. It was pruned to include only high-confidence interactions–i.e. interactions with combined scores of 700 or greater. For each differential expression analysis, the posterior probabilities of differential expression were assigned to their corresponding nodes. Edge weights were calculated as the product of the posterior probabilities of their incident nodes. Thus, edge weights represent the joint posterior probability of differential expression of the interacting proteins. The networks were further pruned to include only edges with weight 0.98 or greater. The giant component of the resulting network is the differentially regulated portion of the protein-protein interaction network. Communities in these networks were identified using the Louvain algorithm implemented in the ‘igraph’ R package^40^. Gene set enrichment of the network communities was calculated using Fisher’s exact test and human Reactome gene sets^41^.

### Analysis of regulation with respect to NAS vs fibrosis

Identification of differential expression that is exclusive to NAS or fibrosis stage was based on the posterior probability of differential expression with respect to each measure. We calculated the posterior probability that a gene i is exclusively regulated with respect to NAS as

$${P}_{i}^{exNAS}={P}_{i}^{NAS}\times (1-{P}_{i}^{Fib})$$

and exclusively regulated with respect to fibrosis stage as

$${P}_{i}^{exFib}={P}_{i}^{Fib}\times (1-{P}_{i}^{NAS})$$

where ${P}_{i}^{NAS}$ is the posterior probability of differential expression with respect to NAS and ${P}_{i}^{Fib}$ the posterior probability of differential expression with respect to fibrosis stage. Gene sets enriched for exclusively regulated genes were identified using the ‘geneSetTest’ function from the ‘limma’ Bioconductor package, which performs a rank-based competitive test^42^.

### Pathway analysis

Additional pathway analyses were performed using the gene set variation method (GSVA) followed by ordinal regression^20^. GSVA generates pathway-level quantifications from gene-level quantifications. To identify differentially expressed pathways, we used the pathway-level quantifications from GSVA in conjunction with the same ordinal regression strategy that was used for the gene-level analysis.

### Calculation of gene-level NAS and fibrosis scores

The ordinal regression models, once fit, can be used to predict the probability of assignment to an ordinal level given the expression value of a gene. We used this feature of the model to assign a score for each gene in every sample–i.e. the gNAS and gFib scores. Specifically, the gene-level scores are the weighted mean of the possible ordinal scores, where the predicted probabilities serve as weights:

$${v}_{ij}({x}_{ij},{m}_{i})=\sum _{s\in \sigma }(s\times {\rm{P}}({S}_{j}=s|{x}_{ij},{m}_{i}))$$

Here, v_ij_ is the gene-level score for a gene (i) in a sample (j); *σ* is the set of all possible ordinal scores; x is the gene expression value; and m is a model fit. To reduce bias in the scores we used a 10-fold fitting procedure, where samples in each holdout set were scored using models fit to the samples in the complementary set. Sample-level scores were derived from gene-level scores by computing the mean of the gene-level scores of the 1000 genes with the greatest coefficient of variation (CV). Genes with the highest CV have the greatest information content since ${\rm{P}}(S=s|{x}_{ij})={\rm{P}}(S=s)$ when gene expression and sample score are independent (i.e. when expression is uninformative), in which case $\mathrm{var}({\hat{v}}_{i})$ is minimized.

### Prediction of sample-level scores from clinical measurements and gene expression

Regularized regression models were fit using the ‘glmnet’ and ‘caret’ packages in R^43,44^. Regularization and, when applicable, mixing parameters were selected using a 10-fold 50-repeat cross-validation on a parameter tuning grid. In the case of lasso regression of sample-level scores against gene abundance, we selected somewhat suboptimal (in terms of resampling statistics) regularization parameters in order to achieve a small predictor set of approximately 20 genes. To assess model performance and generalizability, we performed a 5-fold cross-validation using the regularization parameters found in the previous step. Final estimates of coefficients (and variable importance) were derived from models that were fit to the entire dataset.

### Identification of gene and pathway clusters based on gNAS and gFib scores

To identify gene clusters, the gNAS and gFib scores were standardized for each sample across DEGs (1% FDR). The resulting Z-scores were used as input to the gene shaving algorithm using a 10% shaving rate and an *a priori* selection of four clusters^26^. This yielded two sets of gene clusters: one based on gNAS scores, and the other based on gFib scores. Sample clusters were identified by performing hierarchical clustering on the samples using Euclidean distance and Ward’s linkage method. The resulting dendrogram was cut such that the resulting partition maximized the median silhouette width, and the number of clusters that was produced was greater than 2, but less than 20.

Pathway clusters were identified by first determining which hallmark gene sets^27^ were differentially regulated at a 1% FDR. This was achieved using the previously described GSVA method. Each differentially regulated pathway received a score for each sample that was equal to the mean gNAS or gFib score of the corresponding DEGs (1% FDR). These values were standardized for each sample, and the resulting values were clustered using the same hierarchical clustering method described above.

## Supplementary information

Supplementary figures and tables

Supplementary file 1

Supplementary file 2

Supplementary file 3

Supplementary file 4

Supplementary file 5

## Data Availability

All raw data and relevant metadata are available through the Gene Expression Omnibus, Accession Number GSE130970.
