# Supplementary material for: Gene Expression Predicts Histological Severity and Reveals Distinct Molecular Profiles of Nonalcoholic Fatty Liver Disease
Source: Sci Rep. 2019 Aug 29;9:12541. doi: 10.1038/s41598-019-48746-5 (PMC6715650; doi:10.1038/s41598-019-48746-5)
Supplement: Supplementary file 1 — Supplementary figures and tables [file 41598_2019_48746_MOESM1_ESM.pdf]

GENE EXPRESSION PREDICTS HISTOLOGICAL SEVERITY AND REVEALS DISTINCT MOLECULAR PROFILES OF NONALCOHOLIC FATTY LIVER DISEASE

Stephen A. Hoang PhD[1], Abdul Oseini MD[2], Ryan E. Feaver PhD[1], Banumathi Cole PhD[1], Amon Asgharpour MD[3], Robert Vincent MD PhD[2], Mohammad Siddiqui MD[2], Mark J. Lawson PhD[1], Nathan C. Day[1], Justin M. Taylor[1], Brian R. Wamhoff PhD[1], Faridoddin Mirshahi MS[2], Melissa J Contos MD[2], Michael Idowu MD[2] and Arun J. Sanyal MBBS MD[2]

**Corresponding authors:**	Stephen A. Hoang PhD
Title:	Head of Computational Biology, HemoShear Therapeutics
Address:	501 Locust Avenue<br>Charlottesville, VA 22902
Phone:	(434) 872 0196
Fax:	
Email:	hoang@hemoshear.com

**Senior author:**	Arun J. Sanyal
Title:	Z. Reno Vlahcevic Professor of Medicine, Physiology and Molecular Pathology
Address:	MCV Box 980341<br>Richmond, VA 23298-0341
Phone:	(804) 828 6314
Fax:	(804) 828 2992
Email:	arun.sanyal@vcuhealth.org

Affiliations:
1. HemoShear Therapeutics, LLC, Charlottesville, Virginia
2. Div. of Gastroenterology, Hepatology and Nutrition, Dept. of Internal Medicine, Virginia Commonwealth University School of Medicine, Richmond, VA
3. Div. of Surgical Pathology, Dept. of Pathology, Virginia Commonwealth University School of Medicine, Richmond, VA

Supplementary figure 1

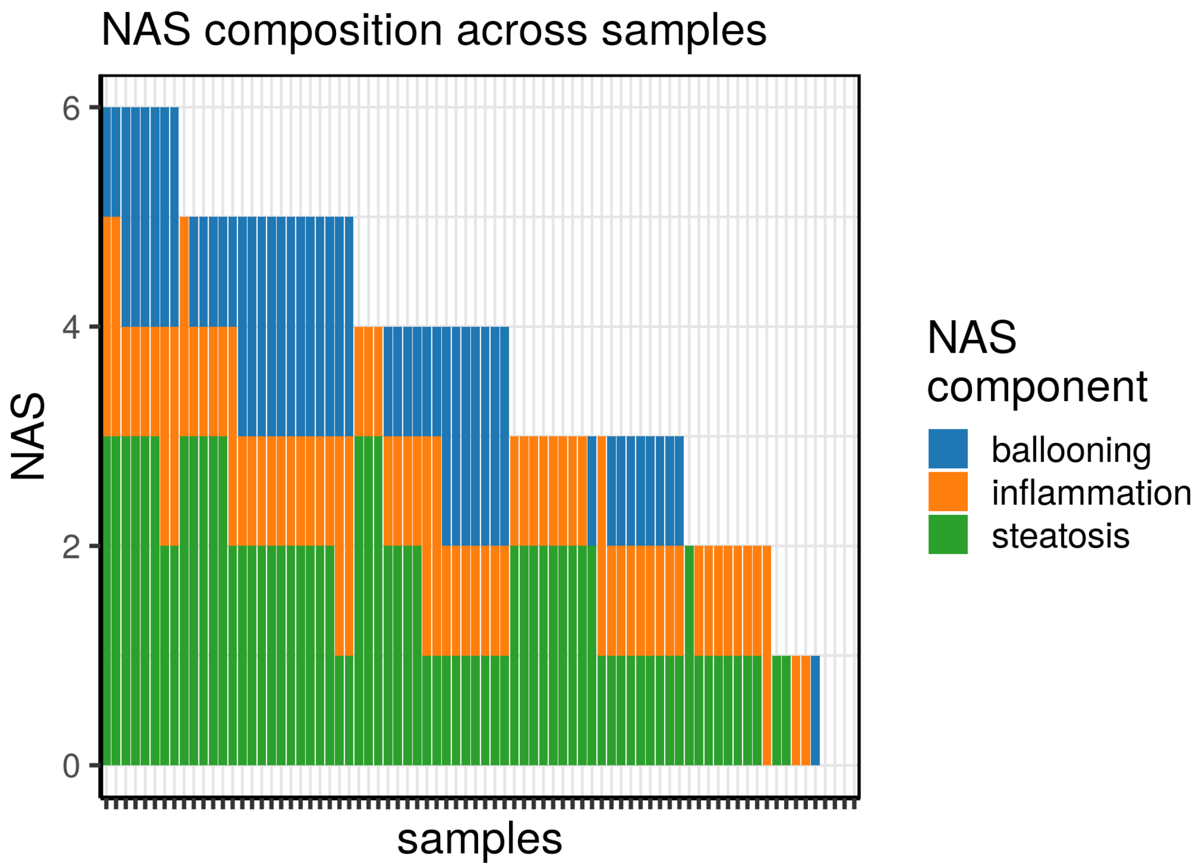

Supplementary figure 1: Composition of the NAFLD activity score (NAS) for each sample. The NAS is the sum score of histological assessments of cytological ballooning (0-2), lobular inflammation (0-3), and steatosis (0-3).

Supplementary figure 2

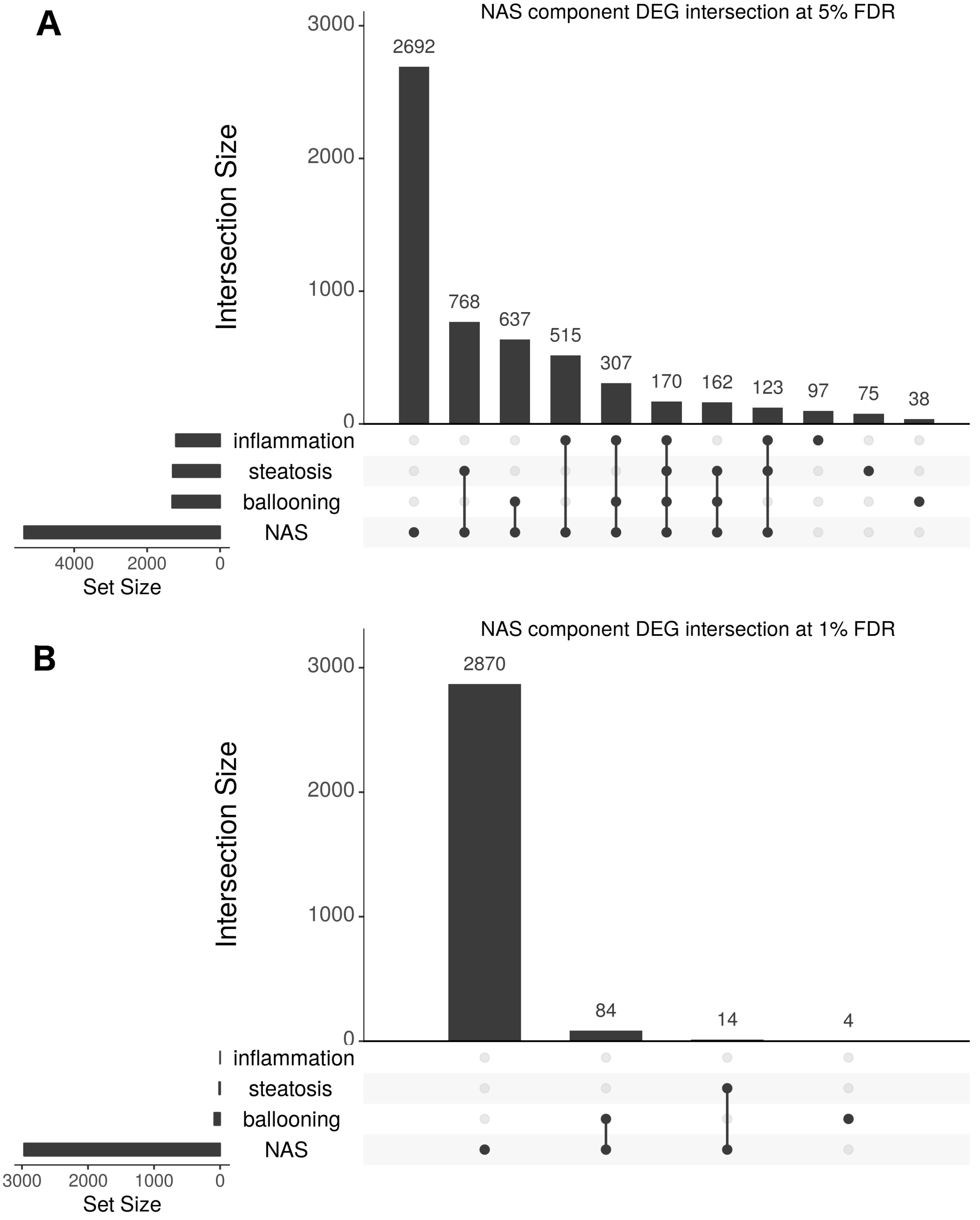

Supplementary Figure 2: Differential expression analysis of NAS components. Plot shows the set intersection cardinality for differentially expressed genes at 5% (A) and 1% (B) FDR thresholds. Differentially expressed genes were assessed with respect to lobular inflammation, steatosis, cytological ballooning, and the composite NAS. Individual set sizes are shown on the

lower left corner of the plots, intersection sizes are on the y-axis, and intersection labels are shown along the x-axis.

Supplementary figure 3

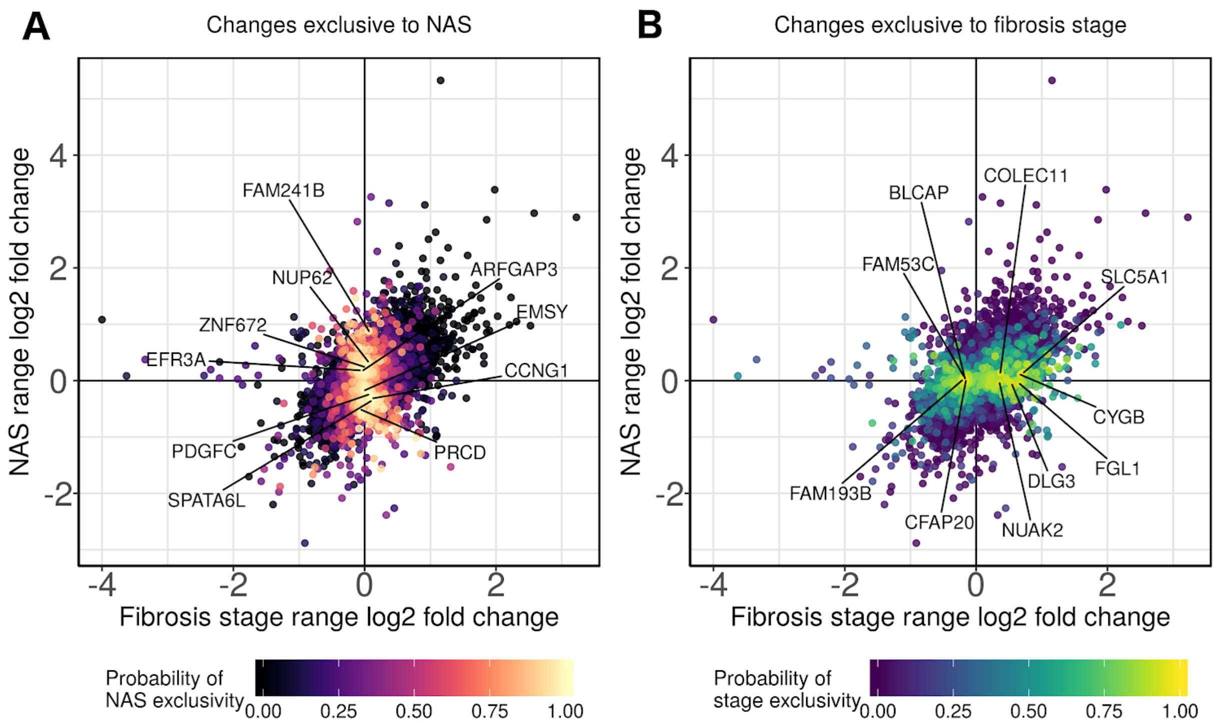

C

Reactome pathways enriched for genes unique to NAS

Reactome set	p-value	FDR
Xenobiotics	0.000400	0.236
Metabolism	0.000412	0.236
Respiratory electron transport	0.000676	0.236
The citric acid (TCA) cycle and respiratory electron transport	0.000692	0.236
Complex I biogenesis	0.000701	0.236
Respiratory electron transport, ATP synthesis by chemiosmotic coupling, and heat production by uncoupling proteins.	0.001356	0.372
Metabolism of lipids	0.001549	0.372
Phospholipid metabolism	0.003338	0.659
Chylomicron assembly	0.004335	0.659
RUNX1 interacts with co-factors whose precise effect on RUNX1 targets is not known	0.007289	0.659

D

Reactome pathways enriched for genes unique to fibrosis stage

Reactome set	p-value	FDR
Peptide chain elongation	1.92e-13	3.23e-10
Eukaryotic Translation Elongation	1.71e-12	9.11e-10
Formation of a pool of free 40S subunits	1.89e-12	9.11e-10
Nonsense Mediated Decay (NMD) independent of the Exon Junction Complex (EJC)	2.16e-12	9.11e-10
Viral mRNA Translation	4.63e-12	1.34e-09
Selenocysteine synthesis	4.78e-12	1.34e-09
Cap-dependent Translation Initiation	6.53e-12	1.37e-09
Eukaryotic Translation Initiation	6.53e-12	1.37e-09
L13a-mediated translational silencing of Ceruloplasmin expression	1.14e-11	2.12e-09
GTP hydrolysis and joining of the 60S ribosomal subunit	2.72e-11	4.58e-09

Supplementary Figure 3: An analysis of gene expression responses that are unique to NAS (A) or fibrosis stage (B). Figures A & B are fold change-fold change plots (NAS vs fibrosis stage), where each point represents a gene, and color represents the posterior probability that a given gene is uniquely differentially regulated with respect to NAS (A) or fibrosis stage (B) (i.e. the

probability approaches one when a gene shows strong evidence of differential regulation in one condition, but not the other). The top 10 genes are labeled in plots A & B. The tables (C & D) show the Reactome gene sets that are enriched for genes with a high degree of uniqueness.

Supplementary Figure 4

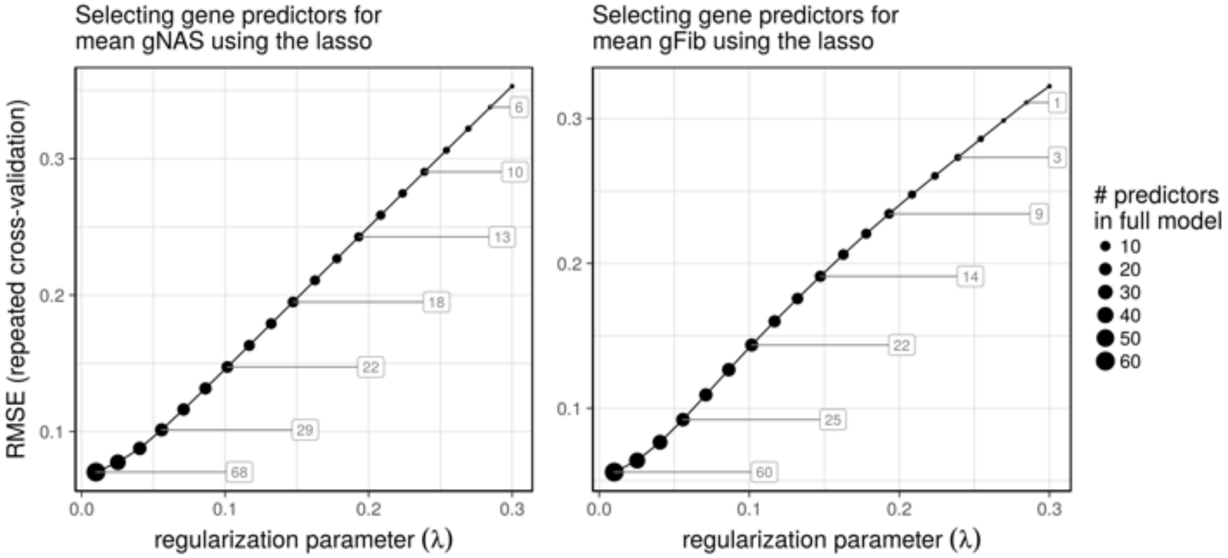

Supplementary Figure 4: The cross-validation root-mean-squared error of the lasso models as a function of the regularization parameter. The number of predictors in each model is represented as point size.

Supplemental Table 1: Characteristics of the populations studied

	Histologically normal control N=6	NAFLD N=72
Age (yrs) mean ± S.D.	49.8 ± 21	52.8 ± 12
Gender (males) n	2	28
Caucasians (n)	5	64
Body Mass Index (kg/m2) mean ± S.D.	25.9 ± 5.8	33.3 ± 5.5
Diabetes (n)	0	29
Hypertension (n)	1	46
Hyperlipidemia (n)	0	42
AST (IU/L) mean ± S.D.	35.7 ± 11	60 ± 40
ALT (IU/L) mean ± S.D.	37.9 ± 12	82 ± 56
Alk Phos (IU/L) mean ± S.D.	96 ± 73	93 ± 33
Bilirubin (mg/dl) mean ± S.D.	0.6 ± 0.2	0.6 ± 0.6
Albumin (gm/dl) mean ± S.D.	4.3 ± 0.2	4.4 ± 0.5
Steatohepatitis (n)	0	58
Steatosis grade: mean	-	2
Lobular inflammation grade: mean	-	1.4
Hepatocellular Ballooning: mean	-	1.8
Fibrosis stage: mean	-	1.3
Distribution of Steatosis grade		
0	6	2
1	-	29
2	-	27
3	-	14
Distribution of Lobular inflammation grade	6	5
0	-	55
1	-	12
2		
Distribution of Hepatocellular ballooning grade		
0	6	14
1	-	28
2	-	30
Distribution of NAS		
0	6	0
1	-	2
2	-	9
3	-	18
4	-	17
5	-	18

6	-	8
Distribution of fibrosis stage		
0	6	19
1	-	28
2	-	9
3	-	14
4	-	2
